# Optimized Hydrophobic Interactions and Hydrogen Bonding at the Target-Ligand Interface Leads the Pathways of Drug-Designing

**DOI:** 10.1371/journal.pone.0012029

**Published:** 2010-08-16

**Authors:** Rohan Patil, Suranjana Das, Ashley Stanley, Lumbani Yadav, Akulapalli Sudhakar, Ashok K. Varma

**Affiliations:** 1 Advanced Centre for Treatment, Research and Education in Cancer, Navi Mumbai, Maharashtra, India; 2 Cell Signaling and Tumor Angiogenesis Laboratory, Boys Town National Research Hospital, Omaha, Nebraska, United States of America; 3 Department of Biomedical Sciences, Creighton University School of Medicine, Omaha, Nebraska, United States of America; 4 Department of Biochemistry and Molecular Biology, University of Nebraska Medical Center, Omaha, Nebraska, United States of America; University of Pennsylvania, United States of America

## Abstract

**Background:**

Weak intermolecular interactions such as hydrogen bonding and hydrophobic interactions are key players in stabilizing energetically-favored ligands, in an open conformational environment of protein structures. However, it is still poorly understood how the binding parameters associated with these interactions facilitate a drug-lead to recognize a specific target and improve drugs efficacy. To understand this, comprehensive analysis of hydrophobic interactions, hydrogen bonding and binding affinity have been analyzed at the interface of c-Src and c-Abl kinases and 4-amino substituted 1H-pyrazolo [3, 4-d] pyrimidine compounds.

**Methodology:**

In-silico docking studies were performed, using Discovery Studio software modules LigandFit, CDOCKER and ZDOCK, to investigate the role of ligand binding affinity at the hydrophobic pocket of c-Src and c-Abl kinase. Hydrophobic and hydrogen bonding interactions of docked molecules were compared using LigPlot program. Furthermore, 3D-QSAR and MFA calculations were scrutinized to quantify the role of weak interactions in binding affinity and drug efficacy.

**Conclusions:**

The in-silico method has enabled us to reveal that a multi-targeted small molecule binds with low affinity to its respective targets. But its binding affinity can be altered by integrating the conformationally favored functional groups at the active site of the ligand-target interface. Docking studies of 4-amino-substituted molecules at the bioactive cascade of the c-Src and c-Abl have concluded that 3D structural folding at the protein-ligand groove is also a hallmark for molecular recognition of multi-targeted compounds and for predicting their biological activity. The results presented here demonstrate that hydrogen bonding and optimized hydrophobic interactions both stabilize the ligands at the target site, and help alter binding affinity and drug efficacy.

## Introduction

Advances in the field of structural biology have provided tremendous opportunities for computational biologists to design small-molecule drug leads with better biological activity and minimal side effects for a disease-specific target. Modern bioinformatics tools could reduce the time needed to prioritize lead compounds. Scientists have been paying more attention to in-silico approaches since the first peptide-based HIV protease inhibitors were developed [1], followed by a target for antihypertension [2], inhibitors of the influenza virus [3] and H5N1 avian influenza [4]–[5], using a structure-based drug design [6]–[10]. Even with such advances, designing a novel anti-cancer drug that works effectively on a patient is still out of reach. Considering the role of protein structures in predicting protein function, we assume that the complexity of each disease can be unraveled if the structure of disease-associated molecules can be accurately visualized in three dimensions at the atomic level. The protein structure provides information about the precise position of each atom and the molecules present in crystallographic forms. This helps in elaborating the location of an active site at the molecular surface of the protein structure where active ligands can be placed.

Weak intermolecular interactions play an important role in stabilizing a ligand energetically at the interface of a protein structure. In this study, we have explored the effect of weak intermolecular interactions on the binding affinity between ligand-protein complexes in order to improve drug efficacy [11]–[13]. Studies of strong covalent bonds, weak hydrophobic interactions and hydrogen bonds raise more questions than have been previously answered [14]. If energetically stabilized drug-like compounds are trapped at the bioactive core of the target site, holding all the biochemical and conformational features, then how is the side effect manifested? How is the binding affinity between drug-ligand complexes associated with drug efficacy? If a suitable environment is provided, are hydrophobic interactions and hydrogen bonds interchangeable? Furthermore, computational biologists (in-silico) are challenged to find supporting factors that bring long-range associated ligand-target complex molecules into small regions where biological activity can be altered. To resolve all these issues, a multi-model approach is needed that explores the dynamic nature of weak intermolecular interactions at the target-drug interface.

**Figure 1:**
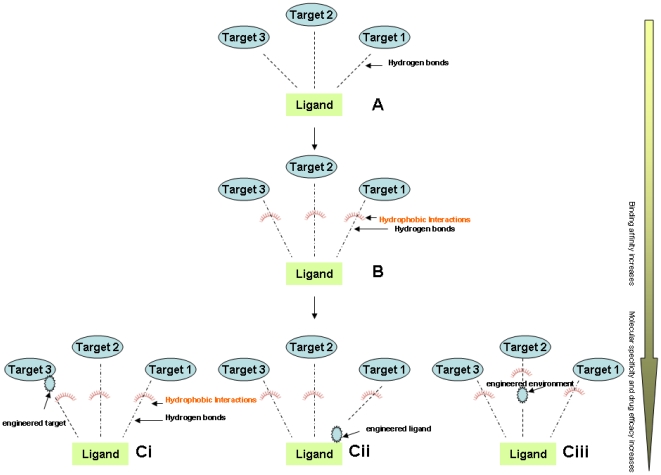
Conformational association of an energetically favored ligand and specific target. The ligand is associated to the specific target with hydrogen bonds and hydrophobic interactions inferring the biological activity of compounds. (**A**) The hypothetical model where the ligand is forming hydrogen bonds to target. (**B**) The ligand is associated with hydrophobic interactions. (Ci) target is modified. (Cii) ligand is modified. (Ciii) ligand is allied with metal-associated hydrogen bonds for a specific target showing the interrelation between long-range hydrogen bonds and hydrophobic interactions.

An increased number of protein structures in the Protein Data Bank have also provided novel opportunities for scientists and clinicians to visualize disease-associated molecules in three dimensions. A drug lead can be designed at the active core of the protein-ligand interface, where the binding affinity is dominated by a small number of atoms [15]–[17]. The predicted hierarchical pathways depicted in **Fig. 1** show in-silico optimization of the role of weak intermolecular interactions between the ligand-target binding affinity and biological activity. This information will encourage scientists to study the interrelationship between hydrophobic interactions and hydrogen bonding when developing new drugs. Most ligands exhibits low binding affinity and thus, they can select the number of targets associated with hydrogen bonds [18] (**Fig. 1A**), however, tight binding is observed if hydrophobic interactions are optimized at the expense of hydrogen bonds [19] (**Fig. 1B**) This schematic representation reveals that low-affinity binders, such as 4-amino substituted pyrimidine derivatives, can be selected and screened for biological activity in a multi-targeted structural environment. Furthermore, incorporation of functional group or metal atoms at the ligand site or target site can increase specificity and binding affinity for one of the target (**Figs 1C i, ii, iii**) even as, major unanswered questions may still need re-evaluation. Does this modified target-drug interface; (1) improve binding affinity, (2) help in finding a specific target, and (3) improve drug efficacy for drug design? To find the answers to these questions focused studies are required to evaluate the role of weak intermolecular interactions in drug discovery. However, finding a suitable target for drug design and development, using structure-based activity relationships, is not a biased approach. Currently, computational biologists face the challenges of designing small molecule inhibitors using experimentally determined structures and improving the biological activity of drug-leads.

To initiate a new approach for drug- design and development, the atomically resolved structure at the ligand-protein interface (i. e, a hot point) is needed [20], In this approach, the positions of a few atoms are stabilized by the number of biophysical parameters such as size, shape, molecular weight, hydrogen bonds, hydrophobic interactions, van der Waals forces and salt bridges [21]–[22]. Hence, any small molecule that binds to a target loses its internal freedom, translational and rotational entropy [23]. After analyzing the interactions that are important for stabilizing the protein-ligand complexes, the focus shifts to the surrounding regions of the hot spot (i.e., the hot regions). Here, the hydrophobic –pocket- associated hot spot and hot regions between 4-amino substituted small molecules and the c-Src [24] and c-Abl [25] protein kinase, have been focused. It is well known that kinases are structurally flexible, thus the dynamic nature of the weak intermolecular interactions of 4-amino-substituted dock molecules at the target-ligand interface can be elaborated.

Kinases are well known for their important role in signal transduction pathways [26]. More than 90 known tyrosine kinases are present in the human genome, Fifty-eight of these are receptor tyrosine kinases and 32 are non-receptor tyrosine kinases. The v-Src protein was the first recognized membrane-bound non-receptor tyrosine kinase. A mutated v-Src protein shows the same characteristics as cellular- Src (c-Src) kinase. Cellular-Src encodes 536 amino acids, whereas Abelson Leukemia viral oncogene homolog 1 (Abl) encodes 1130 amino acids. However, both proteins contain SH3, SH2, and nucleotide- binding functionally important domains.

**Figure 2:**
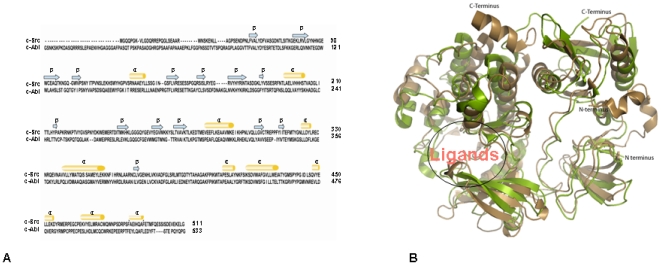
Sequence and structural alignment of c-Src and c-Abl kinase. (**A**) The sequence and secondary structure alignment of c-Src and c-Abl; sequence alignment of c-Src and c-Abl has been carried out using ClustalW. The secondary structure assigned with the SSEA server is depicted as orange cylindrical (alpha helices) and light-blue arrows (beta-strand). (**B**) Three-dimensional structural alignments of c-Src and c-Abl kinases; superimposed structure of c-Src (PDB ID 2H8H) and c-Abl (PDB ID 2FO0); color coding for the ribbon diagram of c-Src is sand yellow and for c-Abl is split-pea-green color. This figure was made using PyMOL program (www.pymol.org).

These bioactive core of c-Src and c-Abl have less sequence similarity (∼45%) and good three dimensional structural resemblance (∼75%) whereas docked 4-amino-substituted compounds have a majority of hydrophobic atoms. This conformationally diversified interface between c-Src and c-Abl protein kinases and 4-amino-substituted dual inhibitors [27] stimulated interest in the optimization of hydrophobic interactions and 3D structural folding at the protein-ligand interface. The inhibitor binding pocket of c-Src and c-Abl is primarily dominated by hydrophobic interactions that play a major role in the binding affinity between proteins and ligands. The comparative structure analysis of both the protein c-Src (PDB ID: 2H8H) and c-Abl (PDB ID: 2FO0) has shown conformational diversity with the rmsd of Cα (carbon-alpha) >10 Å. Despite this change in the rmsd, the overall structural folding at the active core is identical. Since the functional site of the c-Src and c-Abl has a similar folding, it helps the 4-amino substituted inhibitors recognize the binding sites of both the targets. Therefore, it can also be concluded that in addition to low-affinity binding, the protein folding at the biologically active sites should be identical (**Fig. 2A** **and** **Fig. 2B**) if an inhibitor is going to be able to recognize multi-targets. Dual inhibitors or multi-targeted drug leads for a given protein structure can be designed based on: (1) sequence similarity, (2) structural similarity at the active site, (3) binding affinity, and (4) the source of drug lead. Taking all these elements under consideration, efforts were made to understand the drug efficacy by incorporating the hydrophobic interactions at the expense of hydrogen bonding at the target-ligand interface.

## Results and Discussion

Novel classes of disease- modifying drugs, and their associated targets, are very important for studying the structure activity relationship. The presence of a drug lead in proximity to an active pocket of a target site will show a better biological efficacy when compared to a drug lead that is not in close proximity. However, binding affinity of long-range associated target and ligand molecules can be tailored several ways: (1) by optimizing the weak intermolecular interactions, (2) by engineering the active pocket in which the ligand binds, (3) by engineering the pharmacophores or ligand binding directionality, (4) or presence of ions or heavy atoms at the ligand-target interface. Should any of these instances occur, the efficacy of the ligand (a drug-like compound) would be expected to change (**Fig. 1**)? Multi-targeted compounds are low-affinity binders and could become more prominent for a specific target, provided they are exposed to an external conformationally- favored functional group [28]. It has also been reported that binding affinity is a function of the stability of the ligand-target complex formation and further optimize the new bonds that affect the biological activity of a complex molecule. This has been observed recently on dihydropyrancarboxyamides -related zanamivir [29]. Considering these findings, docking studies of 4-amino substituted 1H-pyrazolo [3, 4-d] pyrimidine compounds on c-Src and c-Abl kinases associated with weak hydrophobic interactions, were analyzed. The results presented in this paper provide a novel in-silico based approach to design drug-lead at the hydrophobic core of the protein–ligand interface. The experimentally determined biological activities of inhibitor molecules are align with what has been calculated in-silico.

### Molecular Field Analysis (MFA)

**Figure 3:**
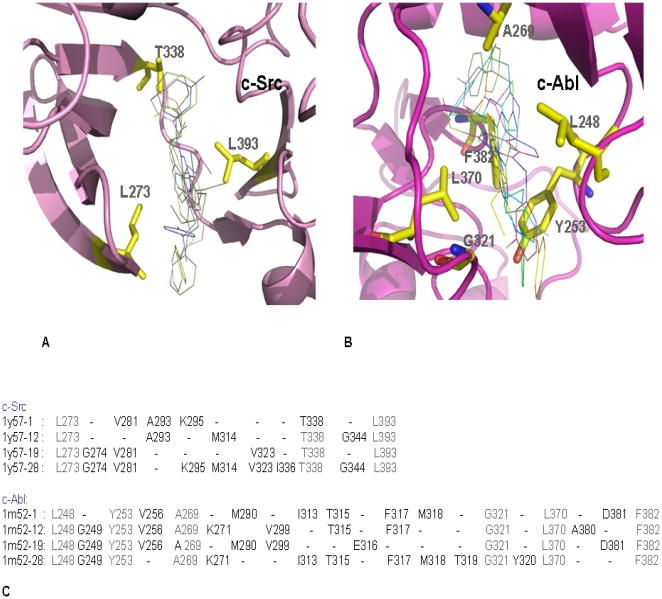
Ligand-associated amino acids of c-Src and c-Abl Kinase. (A) A stick model of active inhibitors 1, 12, 19 and 28 docked in c-Src with conserved hydrophobic amino acids is shown. (**B**) Stick model of active inhibitors 1, 12, 19 and 28 docked in c-Abl with conserved hydrophobic amino acids is shown. (**C**) Alignment of hydrophobic amino acids observed at the interface of c-Src (PDB ID 1Y57), c-Abl (PDB ID 1M52) and functionally important inhibitors as listed in Table S1: (1, 12, 19 and 28). In figure (**B**) and (**C**), structure was aligned using COOT [43] and figure was made by PyMOL program.

Since 1991, the field of drug discovery has used the Quantitative Structure-Activity-Relationship (QSAR) approach in to optimize the drug leads [30]. This method has been successfully used in studies focusing on material molecules, biopolymers, and antigen-antibody interactions [30]–[32]. The QSAR equation was generated using a field value of 700 grid points, with grid dimensions x (−10.883, 7.117), y (−6.401, 11.599), z (−6.520, 5.480), for Molecular Field Analysis (MFA). Thirty-nine compounds of 4-amino-substituted active derivatives were taken as training set and a QSAR equation were generated for c-Src and c-Abl kinase. This QSAR equation was further randomized and cross-validated using the residual data acquired from all the models. We observed a very good agreement between the actual and predicted activity in the unit of Ki and good alignment of all the thirty-nine training set molecules (**Table S1**). In the training set, it is seen that increasing the size of the **R** chain decreases the activity as seen in **2** and **39**, which are less biologically active than compound **28**. The presence of electronegative elements in compounds **19** and **20** shows better activity than in compounds **11** and **13**. In c- Src the bulkier group at the **R2** position seems to decrease in activity. The increase in the size of **R1** shows an increase in activity of c-Src. However, the results are reverse in the case of c-Abl as seen in compound **12**. The quantitative observation of MFA and structural analysis showed that Leu 273, Thr 338 and Leu 393 are amino acids that are conserved in the hydrophobic binding pocket of c-Src, whereas Leu 248, Tyr 253, Ala 269, Gly 321, Leu 370 and Phe 382 are conserved in c-Abl (**Fig. 3A** **and** **Fig. 3B**).

**Figure 4:**
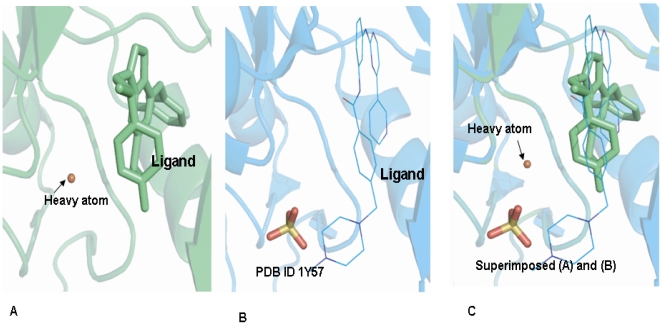
Correlation between crystallographically determined and computationally- generated modeled structure. (**A**) Heavy atom was docked between the ligand and hydrophobic amino acid in c-Src. (**B**) The sulfate group was at the interface of the ligand and hydrophobic amino acid. (**C**) Superimposed of (A) and (B) structure. All three figures were made by PyMOL program.

Based on the QSAR equation, the biological activities of the test set were calculated for 103 compounds. The top 20 best scoring molecules were selected from the top-ranked hit conformations, with a correlation coefficient (r^2^) value 0.936 for c-Src and 0.975 for the c-Abl model. The scoring function of the top 20 test-set molecules is listed in **Table S2**. However in order to compare the conformational changes with the biological activity, this study only analyzed the compounds having high and low scoring values for c-Src and c-Abl. The key amino acids responsible for interactions are mentioned in **Fig. 3C**. A summary of the MFA results is listed in **Table 1**. It was observed from the test set that molecule **4** can be a dual inhibitor, as it showed better biological activity for both c-Src and c-Abl. These results could be due to the presence of para-fluorine at the **R2** position. In c-Src molecule **1**, which has a chlorine phenyl substitution in the **R2** side chain shows optimum biological activity, where as molecule **13** is seen to be the least active compound having the bulky group at **R1** side chain. But in the c-Abl ligand, the occurrence of bromine and fluorine at the **R2** position is the most active. It has been reported that the binding affinity associated with fluorine is remarkably different than the binding affinity associated with other halogens such as Cl, Br, I [11]. Thus, it can be inferred that the halogen substitution at the **R2** position has a profound effect on the K_i_ values. This in-silico prediction for having heavy atom at the ligand–target interface that optimizes hydrophobic interactions and changes the functional activity for a specific target is evidenced by the experimental results. In **Fig. 4A** shows the presence of a docked metal atom, while **Fig. 4B** shows the presence of a crystallographically determined sulfate atom. After superimposing a docked model upon the crystal structure, it has been observed in **Fig. 4C** that the docked atom is located in same part of the crystal structure where the sulfate is positioned. This sulfate is stabilizing the hydrophobic core of the c-Src–ligand complex.

**Table 1:** Summary of MFA results.

MFA results	c-Src	c-Abl
r^2^	0.936	0.975
N_obs_	37.000	36.000
N_vars_	15.000	15.000
LSE(Least square error of fit)	0.283	0.46
R(correlation coefficient)	0.968	0.987
BS r^2^	0.917	0.767
PRESS	47.939	68.594
Dep SD	163.938	66.037
Dep Mean	2.695	0.789

Where r^2^ =  Square of correlation coefficient,

N_obs_ =  Number of observation,

N_vars_  = Number of terms in the equation,

BS r^2^ (Bootstrap r^2^)  =  Average squared correlation coefficient which is calculated during the validation procedure,

PRESS  =  Predicted sum of square,

Dep SD  =  Sum of square deviations of the dependent variable values from their mean.

Dep Mean  =  Mean of the dependent variable values.

### Functional Sites Analysis

The overall rmsd of Cα of the superimposed c-Src (PDB ID 2H8H) and c-Abl (PDB ID 2FO0) structures using the CCP4i program is >10 Å, this indicate that there is no hope of considering one class of chemical groups or drug-like compounds as dual inhibitors. Despite this great change in the rmsd, the protein folding at the functional site of the c-Src and c-Abl has a very good structural similarity, which helps the 4-amino substituted inhibitors recognize the binding sites of both the genes. This functionally important target-ligand orientation has been confirmed by two approaches. In the first approach, the X-ray crystal structure of c-Src (PDB ID 2H8H) and c-Abl (PDB ID: 2FO0) where the ligand for c-Src, quinazoline and c-Abl, 6-(2,6-Dichlorophenyl)-2-{[3-(Hydroxymethyl) Phenyl]amino}- 8-Methylpyrido [2,3-D] Pyrimidin-7 (8H)-One) were binding at the surface groove of proteins was used. The docking of 4-amino substituted compounds was performed onto the ligand removed protein structure. It has been predicted that all the docked ligands are finding the same position where the structurally predetermined molecules are in place. Using this approach, we found that only a predetermined position was sufficient for analyzing the different parameters of enthalpy or entropy of ligands. The second approach involved random docking of the 4-amino -substituted compounds onto the ligand -removed protein structure of c-Src (PDB ID 2H8H) and c-Abl (PDB ID 2FO0). In this approach, we did the docking of the 4-amino substituted compounds in several ways, such as fixing the c-Src and c-Abl protein structure and docking the flexible ligands, or vice-versa. In this method, the Binding Site Protocol generated a number of expected binding sites. We selected the site having the maximum volume and compared it with the ligand-bound crystal structure. In conclusion, the position of the docked inhibitors at c-Src (PDB ID 2H8H) and c-Abl (PDB ID 2FO0) looked identical in both approaches. The biological activity, in the form of binding affinity, was also identical. These results are listed in **Table S2**. Analyzing the atomic orientations of the 4-amino- substituted inhibitors, we observed that docking on structurally- known ligand-bound bioactive sites is more defined than evaluating random docking. Generally, docking transpires on the basis of shape and the complementarity of the ligand and the protein structure. Ligand is free to rotate and translate all over the target sites with fixed discrete parameters such as contact area and active site volume. However, the possibility of getting better conformation and atomic specificity is more precise in first approach than in the second approach, where both the ligands and the targets are dynamic.

Furthermore, for a low affinity -bound small molecule to recognize multi-targets, the structural similarity at the active core of the target sites also plays a crucial role. The superimposed structure of c-Src and c-Abl predicts the difference in overall conformation of a protein structure, but the ligands binding sites for the 4-amino -substituted dual inhibitor look structurally identical (**Fig. 2B**). The structure of the ligand-bound protein at the binding site of the modeled structure, and the crystallographically determined X-ray structure has been compared. An identical structural homology at the binding interface was predicted (**Fig. 4A** **and** **Fig.4B**). The superimposed model and X-ray structure predicted the identical position of the docked molecule at the active site. This was observed from the position of the crystallographically determined sulfate group and the computationally -docked metal ions (**Fig. 4C**). This docked metal ion optimizes hydrophobic interactions at the outlay of hydrogen bonding. These modified hydrophobic interactions enhance the binding affinity and biological activity of complex molecules and help in stabilizing the biochemical environments of target-drug complexes.

### Analysis of Hydrogen Bonds

**Figure 5:**
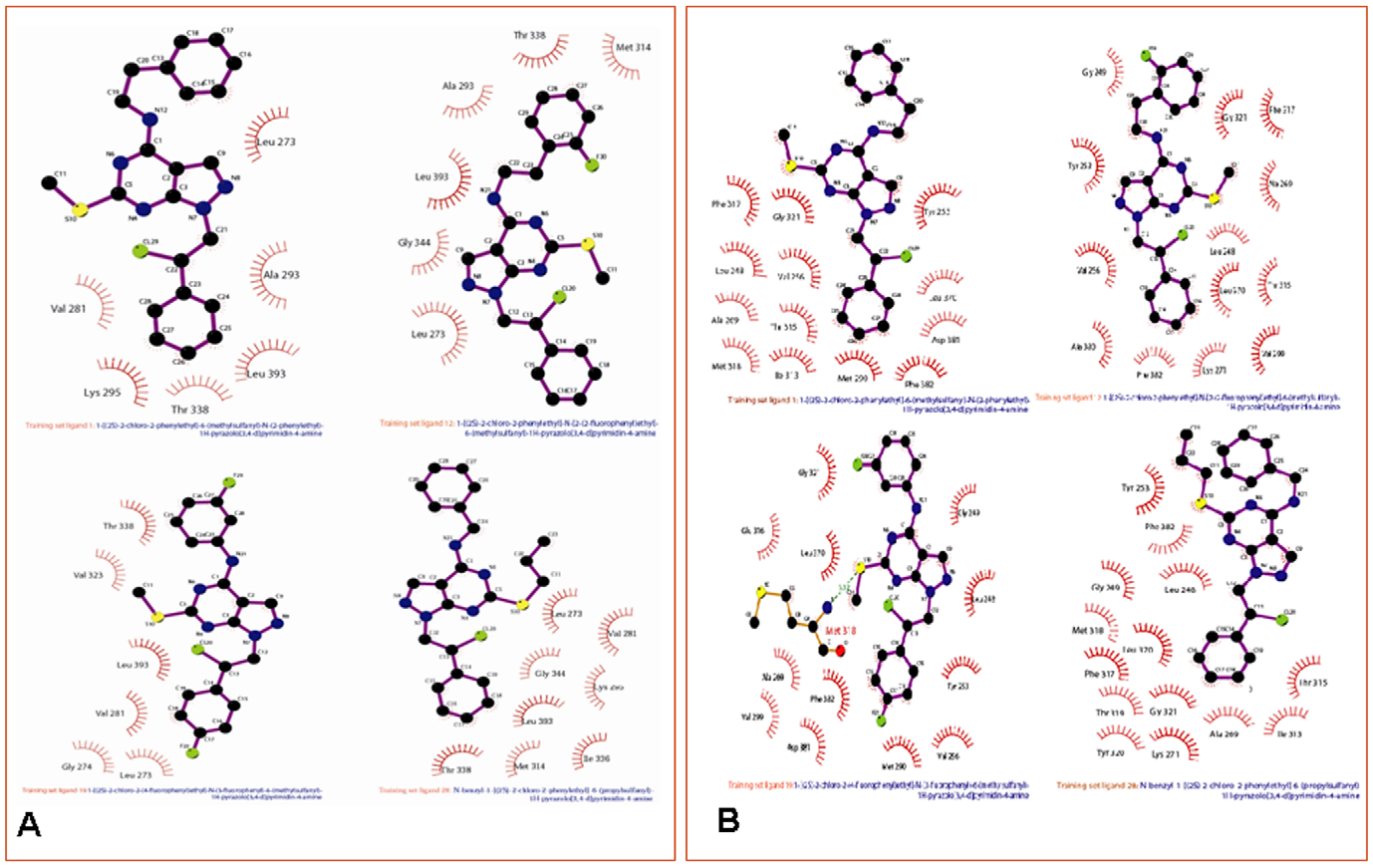
Hydrophobic interactions and hydrogen bonding between ligands and target. (**A**) Two-dimensional representations of interactions observed between c-Src and ligands listed in Table S1: (1, 12, 19 and 28). (**B**) Two-dimensional representations of interactions observed between c-Abl and ligands listed in Table S1: (1, 12, 19 and 28). Hydrogen bonds are depicted with dashed line and hydrophobic interactions are shown as arcs. Both the figures were made using LIGPLOT program.

The importance of hydrogen bonds in the binding affinity of a target-drug has been described extensively [33]. Where as, how hydrogen bonds optimize the hydrophobic interactions, at the protein-ligand interface that increases the binding affinity of complex molecules, has not been properly analyzed. In this paper, we have studied the relative contribution of hydrogen bonds and hydrophobic interaction of the docked molecules of 4-amino- substituted compounds at the binding sites of c-Src and c-Abl. Using LigandFit software, all 39 docked 4-amino-substituted compounds as reported [27] were analyzed for hydrogen bonding at the binding interface of c-Src and c-Abl. The default parameter for the distance between the hydrogen bond donor and the acceptor was 3.2 Å and the donor proton-acceptor angles between 120–180°, were selected, but no hydrogen bonding, such as C-H…O and O-H…O between the docked molecules and the hydrophobic pocket of the c-Src and c-Abl proteins, was observed (**Fig. 5A** **and** **Fig. 5B**). All the hydrogen bonds [33]–[35] and hydrophobic interactions were analyzed using the LigPlot [36]. The same results were also observed in most of the docking software including ZDOCK and CDOCKER. A change in intermolecular interactions was observed using CDOCKER. Minor changes in the conformation and position of ligands at the active site of c-Src and c-Abl were also observed on docked compounds (**1**, **12**, **19** and **28**) using different software like LigandFit, CDOCKER and ZDOCK (**data not shown**). After we found fewer changes in conformation of ligands at the docking sites, we began manually analyzing the hydrogen bonds and observed weak C-H…O interaction with an H…O distance of >1.85 Å. This kind of weak hydrogen bond can be broken and exchanged for another kind of bond, depending upon the chemical environment at the target, ligand and target-ligand interface.

### Hydrophobic Interactions and Functional Activity

The average number of hydrophobic atoms in marketed drugs is 16, with one to two donors and three to four acceptors [37]. This defines the importance of hydrophobic interactions in drug designing. They can increase the binding affinity between target-drug interfaces. It has been already reported that the binding affinity and drug efficacy associated with hydrophobic interactions can be optimized by incorporating them at the site of the hydrogen bonding [38]. However, this approach is not the only leading method for drug designing. Additionally, the presence of water molecules in hydrophobic regions makes this region quite flexible. There is also the probability of engineering either the drug or the target, or the interface between the drug and the target, to increase the binding affinity.

An increase in the number of hydrophobic atoms in the active core of drug -target interface further increases the biological activity of the drug lead. These loosely bound 4-amino substituent's having a majority of hydrophobic atoms, are dual-inhibitors of c-Src and c-Abl. The functional groups of 4-amino substituent's were modified in order to optimize the hydrophobic interactions at the target-drug interface [30]. These optimized hydrophobic interactions also influence the side effects and toxicity, but to what extent? It has been reported that there are certain pathological conditions in which a low-affinity binding drug works efficiently with minimum side effects [39]. The question arises: How is it possible to improve drug efficacy for these low affinity binding ligands using an in-silico approach? Do conformationally- favored heavy atoms play any role in mediating the binding affinity? To test this assumption, we performed Zn, Cd, Fe and Mn metal atom docking on the inhibitor-docked structure of c-Src and c-Abl. We observed that these atoms were sitting in the same part in the crystal structure of c-Src where the crystallographically- determined atom was located (**Fig. 4A**). This atom-docked model shows an increase in both the binding affinity and in the hydrophobic interactions. The sulfate helps in the crystallization of the c-Src protein. It stabilizes the protein complex by mediating the interactions between c-Src and the ligand (**Fig. 4B**). The superimposed structure predicts the reliability of the docked results and the accuracy of the prediction (**Fig. 4C**). In conclusion, these docked atoms help increase the binding affinity of the target-receptor molecules and optimize the hydrophobic interactions by captivating the hydrogen bonding at the hydrophobic core of the complex.

The ClustalW [40] sequence alignment score for c-Src (PDB ID 2H8H) and c-Abl (PDB ID 2FO0) is 38. The SuperPose [41] alignment gives a 42% sequence identity and a 61% sequence similarity, with a gap in sequence of 8.2% similarly. Observing less sequence similarity of the conserved hydrophobic amino acids and the good structural resemblance at the active core of c-Src and c-Abl kinases became focused to understand the role of hydrophobic interactions, binding affinity and the functional efficacy of ligands. We turned T (Turn) to C (coil), G (3_10_ helix) to H (Helix) and removed the B (bridge) to compensate for the secondary structure. SSEA [42] (Secondary Structure Element Alignment, a web-based server) which resulted in a local secondary structure alignment score of 75.62 and a global alignment of 84.058. Furthermore, we observed secondary structure similarities even at the level of 3_10_ helix besides high rmsd and low sequence similarity (**Fig. 2A** **and** **Fig. 2B**). The average rmsd between c-Src (PDB ID: 2H8H) and c-Abl (PDB ID: 2FO0) for Cα using CCP4i [43] is ∼10 Å, and for a side chain is ∼12 Å. The superimposed structure of PDB ID 2H8H (c-Src) and PDB ID 2FO0(c-Abl) showed a conformational divergence at the binding site, with an overall rmsd of Cα 9.65 Å and for a side chain of 9.66 Å. Overall the structural fold at the ligand binding site displayed a similar structural folding (**Fig. 2B**). In this study, efforts have been made to reveal how few organic residues can work as a dual inhibitor in such diversified targets. It is very clear that hydrophobic interactions play a major role in stabilizing the ligands at the binding interface (**Fig. 5A** **and** **Fig. 5B**). A large number of hydrophobic atoms are present in 4-amino substituted compounds and may be important for drug-target binding. This structural environment, in which hydrophobic interactions are optimized at the outlay of hydrogen bonds, can induce a significant change in the biological activity of drug leads. The discovery of influenza neuraminidase inhibitors provided an example where hydrophobic interactions were optimized at the expense of hydrogen bonding [44]. However a further question arises: What could be the source of a heavy atom that can alter the hydrogen bonding into hydrophobic interactions in-vivo? To answer this question, an integrated in-silico, in-vitro, in-vivo effort to study the comparative breakdown of structure at the molecular level is needed.

### Prospects

With fatal diseases, such as cancer, scientists and clinicians are working to discover small molecule anti-cancer compounds. However, the existing approach they are following has not been very successful. Several researchers are screening drug-like compounds using in-silico, in-vitro and in-vivo approaches, but success is still far away. A comparative analysis of binding affinity at the interface of 4-amino substituted compounds and c-Src and c-Abl kinases has revealed that multi-targeted compounds can be better single targeted, if optimized hydrophobic interactions and hydrogen bonding increases its binding affinity. The increase in binding affinity of complex molecules due to optimization of hydrophobic interactions at the target-drug interface, comparatively demonstrate better efficacy of drug leads. This approach would definitely encourage scientists to focus on weak intermolecular interactions, such as hydrophobic interactions, hydrogen bonding and metal-associated interactions for drug design, which have not yet been comprehensively analyzed. The results reported in this paper may help in designing new molecules with potent biological activity and fewer side effects. However, all these predictions are based on the in-silico approach that is not without its drawbacks, deficiencies and limitations of predictive tools which are also always present. Thus the predictions reported in this paper cannot be considered as assets for medicinal counseling until they are further optimized using in-vitro, in-vivo and clinic-based phase trials. Nevertheless, this integrated approach may result in the rational design of potential drugs to fight cancer.

## Materials and Methods

### Selection of Biological Data and Structures

Set of 39 molecules of 4-amino -substituted 1H-pyrazolo [3, 4-d]pyrimidine compounds, along with their biological activity were taken from Schenone et al [27] for training set are listed in **Table S1**. The compounds under study belonged to the following structurally diverse categories; Category A: Linear alkyl amino substituted; Category B: Cyclic amino substituted and Category C: Benzyl amino substituted compounds with fluorine (halogen) at the meta -position.

The biological activity of each compound was compared with Ki, i.e., the inhibitory activity of compounds. Ki value was used to perform the calculations using Accelrys Cerius2 and Discovery Studio software (DS).

### Binding Sites and Binding Affinity Analysis

The binding sites in c-Src and c-Abl protein structures were predicted by using the Binding Site module available in DS. The default parameters in the software for grid size (grid resolution is 0.50 Å, which is the spaces between the grid, 100 points as the minimum binding site, i.e. the minimum number of grid points for the site) were used to predict the binding sites.

The Discovery Studio software module, LigandFit [45] was used to compute the LigScore1 function in order to estimate the binding affinity of the 39 molecules in the training set reported in the units of pKi (-log K_i_). To calculate binding affinity of a test set in pKi, following equation was used in DS: pK_i_ = A+(B vdW) + (C c+pol)−(D BuryPol^2^). LigScore1 is one of the scoring functions of LigandFit. In this equation, there are three descriptors: (1) vDW, Lennard-Jones 6–9 potential (kcal/mol); (2) c+pol, a count of the burried polar surface area between c-Src, c-Abl and small molecule 4-amino substituted inhibitor (Å^2^); and (3) BuryPol^2^, the squared sum of the burried polar surface area of the c-Src, c-Abl and small molecule 4-amino substituted inhibitor molecule (Å^2^). A, B, C and D are constant parameters that obtained through regression analysis.

### Calculation of rmsd and Cavity Volume of Proteins

The CCP4i [46] program was used to calculate the overall rmsd of Cα (carbon-alpha) of the superimposed structure of c-Src (PDB ID 2H8H) and c-Abl (PDB ID 2FO0). The cavity volume at the binding sites was calculated, using DS (Binding Site module) for the coordinates obtained from protein data bank (c-Src, PDB ID: 1YOL, 1Y57, 1FMK, 2H8H and for c-Abl, PDB ID: 1M52 and 2FO0). A binding site having maximum volume was selected manually, as it should have the appropriate volume for a drug to adopt minimal energy. The cavity volume of different proteins is listed in the **Table S3**.

### Molecular Modeling and Docking

All the molecular models were built using Accelrys DS. A CHARMM force field [47] was applied to each model to stabilize the coordinate from thermodynamic environments and to remove some steric clashes and bad contacts between the ligand and target. PDB ID: 1Y57 (the X-ray crystal structure of a human tyrosine-protein kinase c-Src) and 1M52 (the crystal structure of a c-Abl kinase domain) coordinates were used to dock the 39 dual inhibitors [27]. The LigandFit module [45] from Discovery Studio was used to perform the docking, based on shape-based searching and Monte Carlo methods. While docking, the variable trials Monte Carlo conformation was applied where the number of steps depends on the number of rotatable bonds in the ligand. By default, the torsions number is 2, the number of trials is 500 and the maximum successive failure is 120. Furthermore, protein-ligand interactions were evaluated with Ligscore1, hydrogen bonding and vDW functions. The position of docked molecules was also re-evaluated, using ZDOCK and CDOCKER docking software. Different coordinate files (PDB) were selected to confirm the position of dock molecules independently. The rmsd of different docked ligand at the ligand binding modes is given in **Table S4**.

### Molecular Field Analysis (MFA)

Using Cerius2 (Accelrys software Inc) QSAR module, MFA was performed for all the training set as well as the test set molecules, to quantify the interaction energy for a set of 3D structures between the target and the ligand molecules. The field was generated at each grid point (700 points) of the rectangular grid, which is regularly spaced at 2.0 Å with grid dimensions x (−10.883, 7.117), y (−6.401, 11.599), z (−6.520, 5.480). In addition, a number of spatial and structural descriptors such as polarizability, dipole moment, radius of gyration, molecular area, molecular dimension, density, principal moment of inertia, molecular volume, molecular weight, number of rotatable bonds, hydrogen bond donors and acceptors, AlogP, and molar refractivity were also calculated along with the molecular field. Regression analysis was carried out using the genetic partial least squares (G/PLS) method which consists of 5000 generations with a population size of 100. The truncate energy cut off for both steric and electrostatic grid was between −30 to +30 Kcal. Cross-validation was performed with the leave-one–out principle to provide an almost unbiased estimate of the model. The QSAR equation generated by MFA for the 39 molecules of the training set and Scatter-plot of actual versus predicted activity for c-Src and c-Abl are shown in **File S1**. Using this QSAR equation, a test set was generated and the best 20 were selected on the basis of predicted biological activity against c-Src and c-Abl.

## Supporting Information

File S1 The QSAR equation and Scatter-plot of actual versus predicted activity for c-Src and c-Abl.
(0.09 MB DOC)

Table S1 The training set of 39 molecules of 4-amino substituted, with their biological activity and structural alignment.
(0.48 MB DOC)

Table S2 The test set of 20 molecules of 4-amino substituted, with their biological activity.
(0.06 MB DOC)

Table S3 Cavity volume in the protein structures of c-Src and c-Abl selected for docking.
(0.03 MB DOC)

Table S4 RMSD values of ligands (only for the molecules 1, 12, 19 and 28) at binding site of c-Src and c-Abl measured using different algorithms.
(0.04 MB DOC)
