# Supplementary material for: Optimized Hydrophobic Interactions and Hydrogen Bonding at the Target-Ligand Interface Leads the Pathways of Drug-Designing
Source: PLoS One. 2010 Aug 16;5(8):e12029. doi: 10.1371/journal.pone.0012029 (PMC2922327; doi:10.1371/journal.pone.0012029)
Supplement: File S1 — The QSAR equation and Scatter-plot of actual versus predicted activity for c-Src and c-Abl. (0.09 MB DOC) [file pone.0012029.s001.doc]

**QSAR equation of MFA for c-Src, biological activity is in the unit of (Ki)_i_**

**Activity** = 3.26044 + 0.065974 (HO-/475) + 0.035056 (HO-/481) - 0.034831

X (H+/165) - 0.044147 (H+/320) - 0.032136 (CH3/654) + 0.016452 (HO-/334) - 0.079362 (HO-/412) + 0.069275 (CH3/648) + 0.09263 (HO-/243) - 0.087644 X (CH3/475) - 0.058515 (H+/410) - 0.080377 (CH3/458) + 0.02798 (HO-/384) + 0.049331 (CH3/614)

**Probes:** H+, Donar/Acceptor, CH3

r2: 0.936

N_obs_: 37.000

N_vars_: 15.000

LSE: 0.283

r: 0.968

XV r2: 0.708

BS r2 : 0.917

PRESS: 47.939

Dep SD: 163.938

Dep Mean: 2.695

**Scatter-plot of actual vs predicted activity of c-Src**


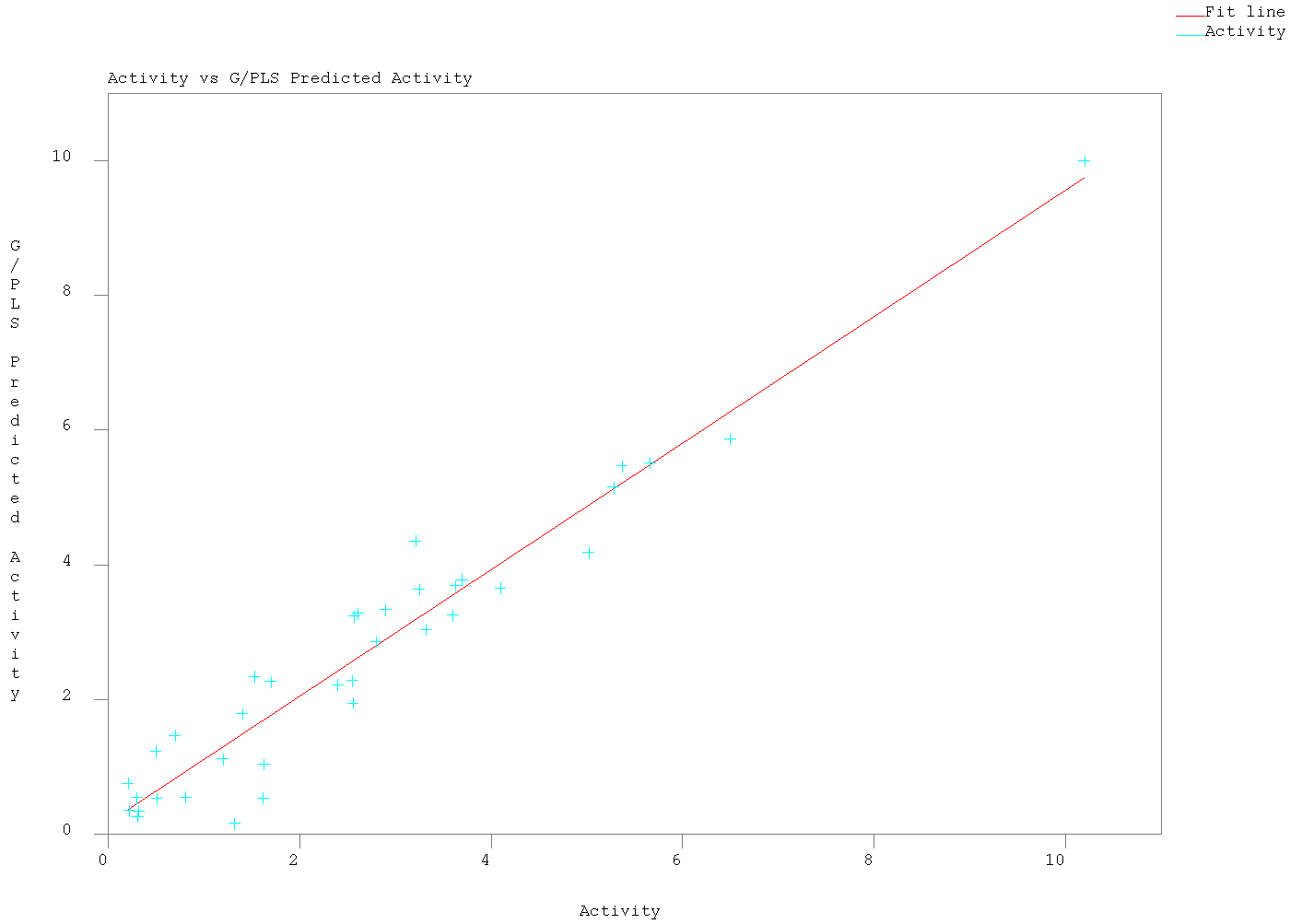


**QSAR equation of MFA for c-Abl, biological activity is in the unit of (Ki)_i_**

**Activity** = 16.0049 - 0.228884 (CH3/318) + 0.000166 (CH3/209) - 0.045315

X (H+/183) + 0.002254 (CH3/587) + 0.001688 (CH3/330) - 0.013869

X (CH3/265) + 0.052064 (H+/295) + 0.026846 (CH3/278) - 0.001152 (CH3/358) - 0.291064 (CH3/474) - 0.253441 (CH3/270) + 0.022238 (H+/339) + 0.003499 X (CH3/187) - 0.110056 (H+/33)

**Probes**: H+, CH3

r^2^: 0.975

N_obs_: 36.000

N_vars_: 15.000

LSE: 0.46

r: 0.987

XV r2: -0.039

BS r2 : 0.767

PRESS: 68.594

Dep SD: 66.037

Dep Mean: 0.789

**Scatter-plot of actual vs predicted activity of c-Abl**

**
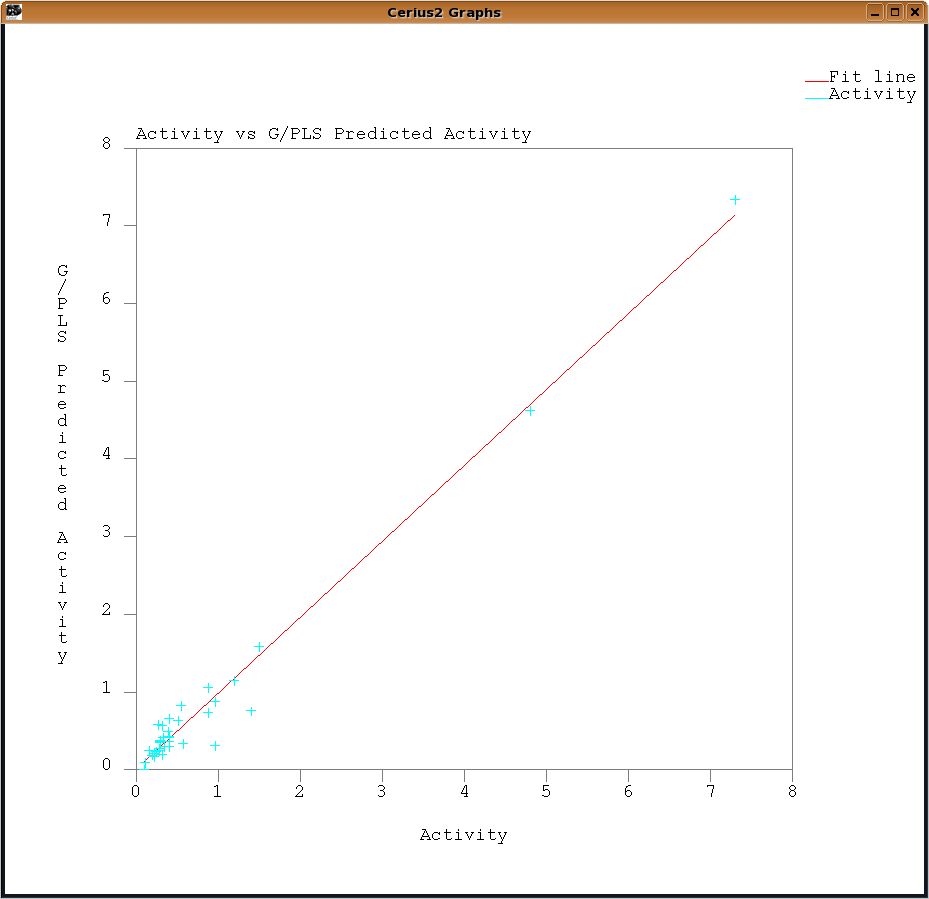
**
