# Supplementary material for: Optimized Hydrophobic Interactions and Hydrogen Bonding at the Target-Ligand Interface Leads the Pathways of Drug-Designing
Source: PLoS One. 2010 Aug 16;5(8):e12029. doi: 10.1371/journal.pone.0012029 (PMC2922327; doi:10.1371/journal.pone.0012029)
Supplement: Table S1 — The training set of 39 molecules of 4-amino substituted, with their biological activity and structural alignment. (0.48 MB DOC) [file pone.0012029.s002.doc]

**Table 1A: The set of 39 molecules of 4-amino substituted, with their biological activity in the unit of ( Ki).**

| **S.**  **No** | **R** | **R^1^** | **R^2^** | **c-Src actual activity**  **Ki(μM)** | **c-Src predicted Ki(μM)**  **Ligscore** | **c-Src predicted activity MFA**  **Ki(μM)** | **c-Abl actual activity**  **Ki(μM)** | **c-Abl predicted Ki(μM)**  **Ligscore** | **c-Abl predicted activity**  **MFA**  **Ki(μM)** |
| --- | --- | --- | --- | --- | --- | --- | --- | --- | --- |
| 1 | SCH_3_ | NHCH_2_CH_2_C_6_H_5_ | CH_2_-CHCl-C_6_H_5_ | 0.7+ 0.2 | 0.537 | 1.467 | 7.3+ 0.2 | 2.344 | 7.339 |
| 2 | SCH_3_ | NHCH_2_C_6_H_5_ | CH_2_-CHCl-C_6_H_5_ | 3.7+ 0.9 | 4.467 | 3.782 | 0.3 +0.1 | 0.309 | 0.38 |
| 3 | SCH_3_ | NHC_3_H_7_ | CH_2_-CHCl-C_6_H_5_ | 2.9+ 0.8 | 1.35 | 3.334 | 4.8 + 0.6 | 2.041 | 4.623 |
| 4 | SCH_3_ | NHC_4_H_9_ | CH_2_-CHCl-C_6_H_5_ | 1.7+ 0.4 | 2.188 | 2.268 | 1.2 + 0.3 | 2.455 | 1.148 |
| 5 | SCH_3_ | NH(CH_2_)_2_OC_2_H_5_ | CH_2_-CHCl-C_6_H_5_ | NA | 0.38 | 2.21 | 1.5+ 0.5 | 1.621 | 1.583 |
| 6 | SCH_3_ | 1-Pyrrolidinyl | CH_2_-CHCl-C_6_H_5_ | ND | 0.091, 0.083, 0.112, 2.238 | 0.551 | NA | 0.048, 0.123, 0.162, 0.295, 0.38 | 0.412 |
| 7 | SCH_3_ | 1-Piperidinyl | CH_2_-CHCl-C_6_H_5_ | 2.4 + 0.7 | 1.148, 2.188 | 2.221 | NA | 0.068, 0.117, 0.28 | 0.815 |
| 8 | SCH_3_ | 4-Morpholinyl | CH_2_-CHCl-C_6_H_5_ | 6.5+ 1.0 | 2.344 | 5.87 | NA | 0.029, 0.051, 0.079, 0.081, 0.174 | 2.905 |
| 9 | SCH_3_ | N(C_2_H_5_)_2_ | CH_2_-CHCl-C_6_H_5_ | 0.5+ 0.1 | 0.38 | 1.234 | 0.40+ 0.05 | 0.302 | 0.422 |
| 10 | SCH_3_ | NHC_6_H_5_ | CH_2_-CHCl-C_6_H_5_ | 1.2+ 0.3 | 1.58 | 1.114 | 0.40+0.07 | 0.407 | 0.416 |
| 11 | SCH_3_ | NHC_6_H_4_-*m*F | CH_2_-CHCl-C_6_H_5_ | 1.4+ 0.3 | 1.698 | 1.79 | 0.4+ 0.1 | 0.512 | 0.297 |
| 12 | SCH_3_ | NHCH_2_CH_2_C_6_H_4_-*o*F | CH_2_-CHCl-C_6_H_5_ | 10.2+ 1.8 | - | 10.003 | 0.4+ 0.1 | 0.309 | 0.662 |
| 13 | SCH_3_ | NHC_3_H_7_ | CH_2_-CHCl-C_6_H_4_-*p*F | 2.61+ 1.2 | 2.69 | 3.279 | 0.57+ 0.1 | 0.562 | 0.334 |
| 14 | SCH_3_ | NHC_4_H_9_ | CH_2_-CHCl-C_6_H_4_-*p*F | 5.02+ 0.9 | 2.398 | 4.18 | 0.11+0.02 | 0.115 | 0.091 |
| 15 | SCH_3_ | 4-Morpholinyl | CH_2_-CHCl-C_6_H_4_-*p*F | 3.32+ 1.0 | 1.698 | 3.036 | 0.97+ 0.3 | 0.03 | 0.318 |
| 16 | SCH_3_ | NHCH_2_C_6_H_4_-*p*F | CH_2_-CHCl-C_6_H_4_-*p*F | 0.31+ 0.07 | 0.339 | 0.263 | 0.22+ 0.03 | 0.199 | 0.17 |
| 17 | SCH_3_ | NHCH_2_C_6_H_4_-*o*F | CH_2_-CHCl-C_6_H_4_-*p*F | 0.30+ 0.06 | 0.372 | 0.54 | 0.34+ 0.02 | 0.295 | 0.229 |
| 18 | SCH_3_ | NHCH_2_CH_2_C_6_H_5_ | CH_2_-CHCl-C_6_H_4_-*p*F | 3.25+ 0.8 | 1.122 | 3.639 | 0.20+ 0.04 | 0.245 | 0.189 |
| 19 | SCH_3_ | NHC_6_H_4_-*m*F | CH_2_-CHCl-C_6_H_4_-*p*F | 0.21+ 0.02 | 0.033 | 0.755 | 0.22+ 0.04 | 0.282 | 0.212 |
| 20 | SCH_3_ | NHC_3_H_7_ | CH_2_-CHCl-C_6_H_4_-*p*Cl | 1.32+ 0.4 | 2.04 | 0.157 | 0.40+ 0.03 | 0.436 | 0.368 |
| 21 | SCH_3_ | NHC_4_H_9_ | CH_2_-CHCl-C_6_H_4_-*p*Cl | 1.53+ 0.6 | 2.754 | 2.341 | 0.39+ 0.06 | 0.38 | 0.49 |
| 22 | SCH_3_ | N(C_2_H_5_)_2_ | CH_2_-CHCl-C_6_H_4_-*p*Cl | 1.62+ 0.5 | 1.318 | 0.534 | 0.30+ 0.1 | 0.324 | 0.358 |
| 23 | SCH_3_ | 4-Morpholinyl | CH_2_-CHCl-C_6_H_4_-*p*Cl | 3.21+ 0.7 | 4.169 | 4.352 | 0.50+ 0.05 | 0.562 | 0.35 |
| 24 | SC_2_H_5_ | N(C_2_H_5_)_2_ | CH_2_-CHCl-C_6_H_5_ | 5.28+ 0.5 | 1.95 | 5.152 | 0.33+ 0.1 | 0.338 | 0.413 |
| 25 | SC_3_H_7_ | NHC_3_H_7_ | CH_2_-CHCl-C_6_H_5_ | 3.60+0.4 | 2.69 | 3.253 | 0.25+ 0.04 | 0.28, 0.229 | 0.235 |
| 26 | SC_3_H_7_ | NHC_4_H_9_ | CH_2_-CHCl-C_6_H_5_ | 2.56+ 0.7 | 1.288 | 1.943 | 0.52+ 0.08 | 0.512, 0.589 | 0.635 |
| 27 | SC_3_H_7_ | N(C_2_H_5_)_2_ | CH_2_-CHCl-C_6_H_5_ | 5.37+ 0.9 | 0.112 | 5.467 | 0.28+ 0.01 | 0.331 | 0.277 |
| 28 | SC_3_H_7_ | NHCH_2_C_6_H_5_ | CH_2_-CHCl-C_6_H_5_ | 0.51+ 0.1 | 0.977 | 0.527 | 0.10+ 0.01 | 0.115 | 0.008 |
| 29 | SCH_3_ | NHC_3_H_7_ | CH_2_-CHBr-C_6_H_5_ | 2.55+ 0.5 | 3.16 | 2.278 | 0.88+ 0.2 | 0.87 | 0.735 |
| 30 | SCH_3_ | NHC_4_H_9_ | CH_2_-CHBr-C_6_H_5_ | 0.81+ 0.3 | 0.708 | 0.548 | 0.32+ 0.04 | 0.257 | 0.573 |
| 31 | SCH_3_ | NH(CH_2_)_2_OC_2_H_5_ | CH_2_-CHBr-C_6_H_5_ | 5.66+ 0.9 | 2.511 | 5.516 | 0.55+ 0.04 | 0.447 | 0.823 |
| 32 | SCH_3_ | 1-Pyrrolidinyl | CH_2_-CHBr-C_6_H_5_ | 1.63+ 0.4 | 1.412 | 1.037 | 1.40+ 0.3 | 0.316 | 0.756 |
| 33 | SCH_3_ | 1-Piperidinyl | CH_2_-CHBr-C_6_H_5_ | 0.32+ 0.03 | 0.269 | 0.336 | 0.88+ 0.4 | 0.977 | 1.058 |
| 34 | SCH_3_ | 4-Morpholinyl | CH_2_-CHBr-C_6_H_5_ | 2.57+ 0.7 | 1.995 | 3.236 | 0.97+ 0.3 | 0.759 | 0.884 |
| 35 | SCH_3_ | NHCH_2_C_6_H_5_ | CH_2_-CHBr-C_6_H_5_ | 0.22+ 0.01 | 0.123 | 0.348 | 0.19+ 0.03 | 0.151 | 0.685 |
| 36 | SCH_3_ | NHCH_2_CH_2_C_6_H_5_ | CH_2_-CHBr-C_6_H_5_ | 2.80+ 0.4 | 3.09 | 2.858 | 0.27+0.03 | 0.17, 0.525 | 0.584 |
| 37 | N(CH_3_)_2_ | NHC_3_H_7_ | CH_2_-CHCl-C_6_H_5_ | 4.10+ 0.5 | 0.079 | 3.655 | 0.32+0.04 | 0.339 | 0.197 |
| 38 | N(CH_3_)_2_ | 4-Morpholinyl | CH_2_-CHCl-C_6_H_5_ | 3.63+ 0.4 | 3.548 | 3.694 | 0.28+ 0.02 | 0.295 | 0.252 |
| 39 | N(CH_3_)_2_ | NHCH_2_C_6_H_5_ | CH_2_-CHCl-C_6_H_5_ | 4.22+ 0.5 | 3.236 | 3.725 | 0.16+ 0.01 | 0.191 | 0.251 |

**Table 1B: The test set of 20 molecules of 4-amino substituted, with their biological activity**

| **S.No** | **R** | **R^1^** | **R^2^** | **c-Src predicted Ki(μM)**  **Ligscore** | **c-Src predicted MFA**  **Ki(μM)** | **c-Abl Predicted Ki(μM)**  **Ligscore** | **c-Abl predicted MFA Ki(μM)** |
| --- | --- | --- | --- | --- | --- | --- | --- |
| 1 | SC_2_H_5_ | NHC_4_H_9_ | CH_2_-CHCl-C_6_H_5_-pCl | 0.032 | 0.021 | 0.851 | 2.432 |
| 2 | SC_2_H_5_ | NHC_4_H_9_ | CH_2_-CHCl-C_6_H_5_-pF | 0.098 | 0.072 | 0.871 | 2.517 |
| 3 | SC_2_H_5_ | NHC_4_H_9_ | CH_2_-CHCl-C_6_H_5_-pBr | 0.093 | 0.093 | 0.724 | 2.475 |
| 4 | SCH_3_ | NHC_6_H_5_ | CH_2_-CHF-C_6_H_5_ | 0.537 | 0.753 | 0.525 | 0.465 |
| 5 | SCH_3_ | NHC_6_H_4_-*m*F | CH_2_-CHF-C_6_H_5_ | 1.584 | 1.958 | 0.347 | 0.319 |
| 6 | SCH_3_ | NHCH_2_CH_2_C_6_H_4_-*o*F | CH_2_-CHF-C_6_H_5_ | 2.137 | 1.626 | 0.331 | 0.344 |
| 7 | SCH_3_ | NHC_6_H_5_ | CH=CH-C_6_H_5_ | 1.38 | 2.2 | 0.479 | 0.623 |
| 8 | SCH_3_ | NHC_6_H_4_-*m*F | CH=CH-C_6_H_5_ | 1.023 | 1.815 | 0.316 | 0.464 |
| 9 | SCH_3_ | NHCH_2_CH_2_C_6_H_4_-*o*F | CH=CH-C_6_H_5_ | NF | 2.363 | 0.132 | 0.522 |
| 10 | SCH_3_ | 1-piperidino | CH_2_-CHF-C_6_H_5_ | 1.202, 4.07 | 2.575 | 0.646 | 0.684 |
| 11 | SCH_3_ | N(C_2_H_5_) | CH_2_-CHF-C_6_H_5_ | 1.41 | 1.173 | 0.676 | 0.634 |
| 12 | SCH_3_ | NHC_6_H_4_-*p*F | CH_2_-CHBr-C_6_H_5_ | 3.31 | 2.351 | 0.331 | 0.26 |
| 13 | SCH_3_ | NHCH_2_CH_2_C_6_H_4_-*m*F | CH_2_-CHBr-C_6_H_5_ | 9.54 | 2.556 | 0.331 | 0.318 |
| 14 | SCH_3_ | NHC_6_H_4_-*p*F | CH_2_-CHCl-C_6_H_5_ | 1.778 | 2.229 | 0.234 | 0.251 |
| 15 | SCH_3_ | NHC_6_H_4_-*o*F | CH_2_-CHF-C_6_H_5_-pF | 2.454 | 2.402 | 0.132 | 0.184 |
| 16 | SCH_3_ | NHCH_2_C_6_H_4_-*m*F | CH_2_-CHF-C_6_H_5_-pF | 1.097 | 1.113 | 0.389 | 0.207 |
| 17 | SCH_3_ | NHC_6_H_4_-*p*F | CH_2_-CHBr-C_6_H_5_-pF | 0.851 | 0.919 | 0.007 | 0.001 |
| 18 | SCH_3_ | NHCH_2_C_6_H_4_-*m*F | CH_2_-CHCl-C_6_H_5_ | 1.738 | 2.645 | 0.324 | 0.378 |
| 19 | SCH_3_ | NHCH_2_CH_2_C_6_H_4_-*m*F | CH_2_-CHCl-C_6_H_5_ | 1.514 | 2.512 | 0.234 | 0.305 |
| 20 | SCH_3_ | NHCH_2_CH_2_C_6_H_4_-*m*F | CH_2_-CHF-C_6_H_5_ | 3.02 | 1.574 | 0.178, 0.708 | 0.35 |

**Table 1C: Cavity volume in the protein structures selected for docking**

| Protein Name | PDB ID | Resolution ( Å) | Binding Site Volume (Å^3^) |
| --- | --- | --- | --- |
| c-Src | 1YOL | 2.3 | 243.875 |
|  | 1FMK | 1.5 | 338.125 |
|  | 2H8H | 2.2 | 295.625 |
| c-Abl | 1M52 | 2.6 | 315.25 |
|  | 2FO0 | 2.27 | 262.000 |

**Table 1D: RMSD values of ligand binding modes measured using different algorithms**

| Molecules | | LigandFit-ZDOCK | LigandFit-CDOCKER | ZDOCK-CDOCKER |
| --- | --- | --- | --- | --- |
| Src | 1 | 0.5600 | 0.1408 | 0.545 |
|  | 12 | 0.8962 | 0.1781 | 0.8545 |
|  | 19 | 0.5451 | 0.1601 | 0.4961 |
|  | 28 | 0.4093 | 0.8603 | 0.8685 |
| Abl | 1 | 0.9164 | 0.3141 | 1.0745 |
|  | 12 | 0.5296 | 0.9292 | 0.8167 |
|  | 19 | 0.6847 | 0.8748 | 0.4985 |
|  | 28 | 0.7731 | 0.1892 | 0.7989 |

**QSAR equation of MFA for c-Src K_i_**

**Activity** = 3.26044 + 0.065974 (HO-/475) + 0.035056 (HO-/481) - 0.034831

X (H+/165) - 0.044147 (H+/320) - 0.032136 (CH3/654) + 0.016452 (HO-/334) - 0.079362 (HO-/412) + 0.069275 (CH3/648) + 0.09263 (HO-/243) - 0.087644 X (CH3/475) - 0.058515 (H+/410) - 0.080377 (CH3/458) + 0.02798 (HO-/384) + 0.049331 (CH3/614)

**Probes:** H+, Donar/Acceptor, CH3

r2: 0.936

N_obs_: 37.000

N_vars_: 15.000

LSE: 0.283

r: 0.968

XV r2: 0.708

BS r2 : 0.917

PRESS: 47.939

Dep SD: 163.938

Dep Mean: 2.695


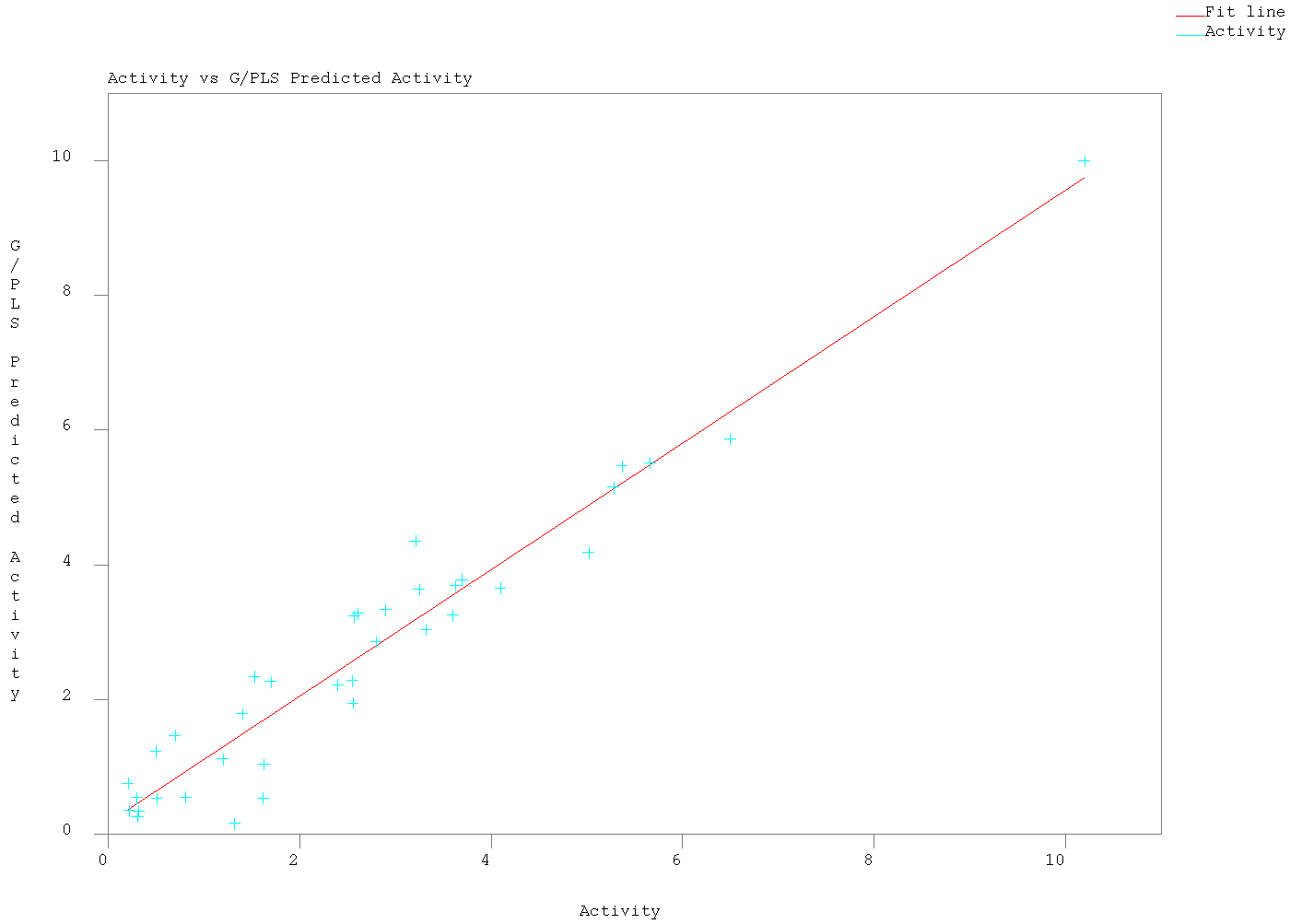


**Graph 1:** Actual vs predicted activity of c-Src

**QSAR equation of MFA for c-Abl K_i_**

**Activity** = 16.0049 - 0.228884 (CH3/318) + 0.000166 (CH3/209) - 0.045315

X (H+/183) + 0.002254 (CH3/587) + 0.001688 (CH3/330) - 0.013869

X (CH3/265) + 0.052064 (H+/295) + 0.026846 (CH3/278) - 0.001152 (CH3/358) - 0.291064 (CH3/474) - 0.253441 (CH3/270) + 0.022238 (H+/339) + 0.003499 X (CH3/187) - 0.110056 (H+/33)

**Probes**: H+, CH3

r^2^: 0.975

N_obs_: 36.000

N_vars_: 15.000

LSE: 0.46

r: 0.987

XV r2: -0.039

BS r2 : 0.767

PRESS: 68.594

Dep SD: 66.037

Dep Mean: 0.789


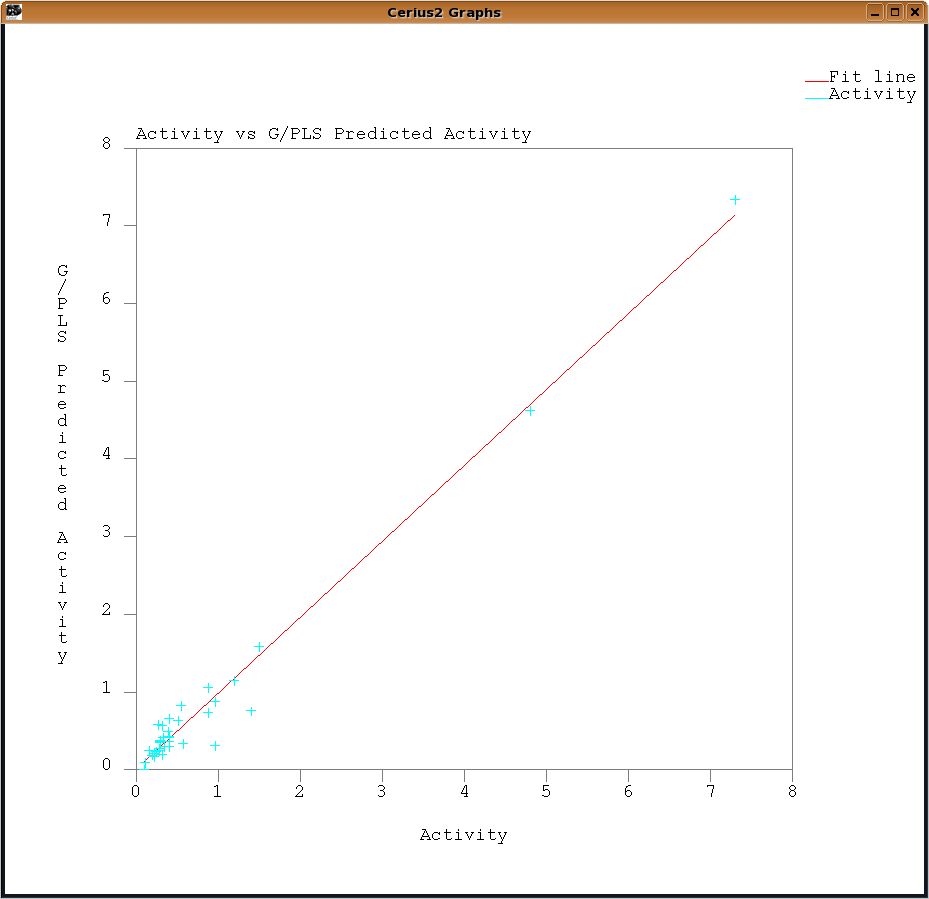


**Graph 2**: Actual vs predicted activity of c-Abl


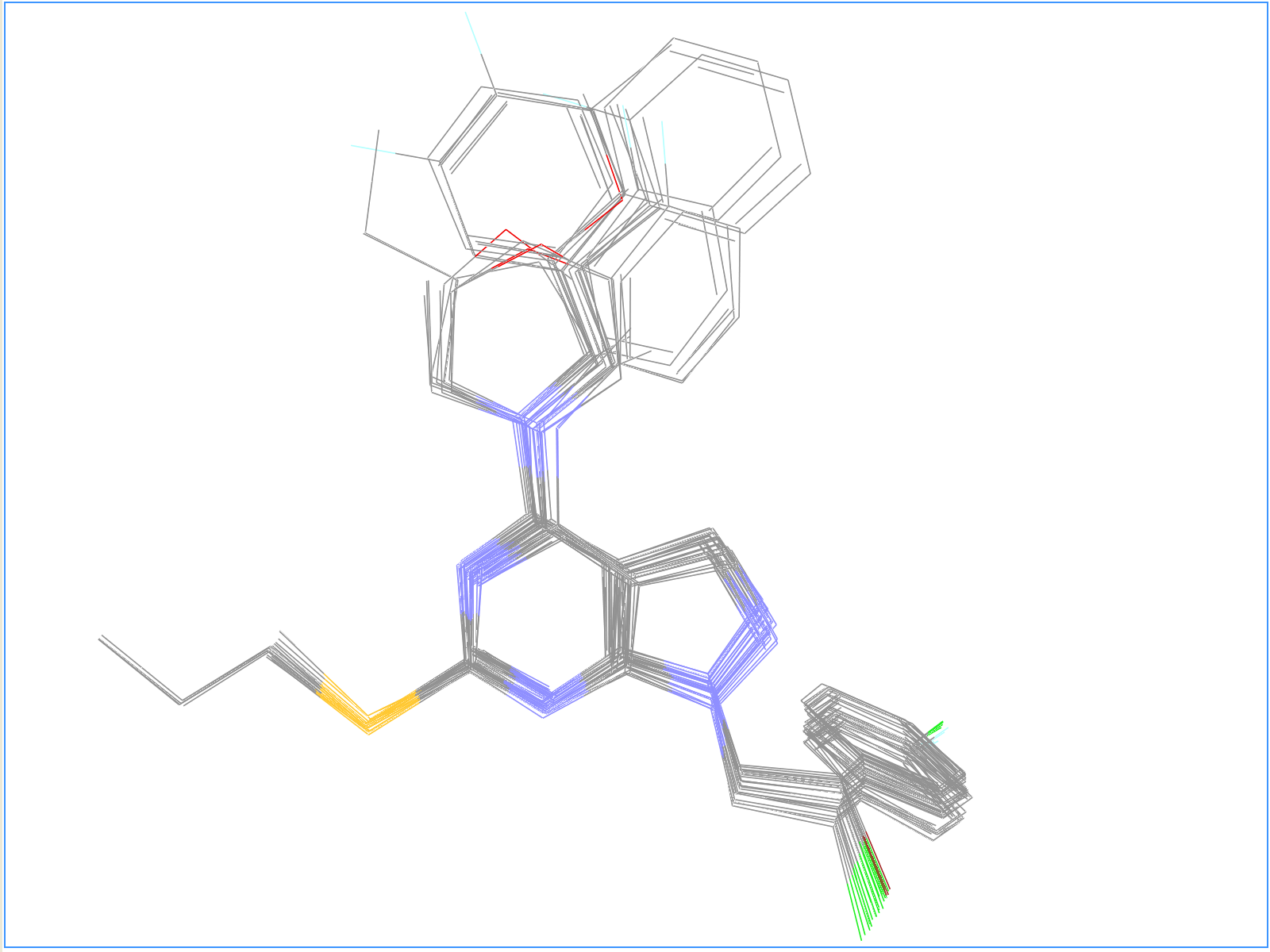


Alignment of 39 training set molecules (listed on Table 1)
