# Supplementary material for: Optimized Hydrophobic Interactions and Hydrogen Bonding at the Target-Ligand Interface Leads the Pathways of Drug-Designing
Source: PLoS One. 2010 Aug 16;5(8):e12029. doi: 10.1371/journal.pone.0012029 (PMC2922327; doi:10.1371/journal.pone.0012029)
Supplement: Table S2 — The test set of 20 molecules of 4-amino substituted, with their biological activity. (0.06 MB DOC) [file pone.0012029.s003.doc]

**Table S2: The test set of 20 molecules of 4-amino substituted, with their biological activity**

| **S.No** | **R** | **R^1^** | **R^2^** | **c-Src predicted Ki(μM)**  **Ligscore** | **c-Src predicted MFA**  **Ki(μM)** | **c-Abl Predicted Ki(μM)**  **Ligscore** | **c-Abl predicted MFA Ki(μM)** |
| --- | --- | --- | --- | --- | --- | --- | --- |
| 1 | SC_2_H_5_ | NHC_4_H_9_ | CH_2_-CHCl-C_6_H_5_-pCl | 0.032 | 0.021 | 0.851 | 2.432 |
| 2 | SC_2_H_5_ | NHC_4_H_9_ | CH_2_-CHCl-C_6_H_5_-pF | 0.098 | 0.072 | 0.871 | 2.517 |
| 3 | SC_2_H_5_ | NHC_4_H_9_ | CH_2_-CHCl-C_6_H_5_-pBr | 0.093 | 0.093 | 0.724 | 2.475 |
| 4 | SCH_3_ | NHC_6_H_5_ | CH_2_-CHF-C_6_H_5_ | 0.537 | 0.753 | 0.525 | 0.465 |
| 5 | SCH_3_ | NHC_6_H_4_-*m*F | CH_2_-CHF-C_6_H_5_ | 1.584 | 1.958 | 0.347 | 0.319 |
| 6 | SCH_3_ | NHCH_2_CH_2_C_6_H_4_-*o*F | CH_2_-CHF-C_6_H_5_ | 2.137 | 1.626 | 0.331 | 0.344 |
| 7 | SCH_3_ | NHC_6_H_5_ | CH=CH-C_6_H_5_ | 1.38 | 2.2 | 0.479 | 0.623 |
| 8 | SCH_3_ | NHC_6_H_4_-*m*F | CH=CH-C_6_H_5_ | 1.023 | 1.815 | 0.316 | 0.464 |
| 9 | SCH_3_ | NHCH_2_CH_2_C_6_H_4_-*o*F | CH=CH-C_6_H_5_ | NF | 2.363 | 0.132 | 0.522 |
| 10 | SCH_3_ | 1-piperidino | CH_2_-CHF-C_6_H_5_ | 1.202, 4.07 | 2.575 | 0.646 | 0.684 |
| 11 | SCH_3_ | N(C_2_H_5_) | CH_2_-CHF-C_6_H_5_ | 1.41 | 1.173 | 0.676 | 0.634 |
| 12 | SCH_3_ | NHC_6_H_4_-*p*F | CH_2_-CHBr-C_6_H_5_ | 3.31 | 2.351 | 0.331 | 0.26 |
| 13 | SCH_3_ | NHCH_2_CH_2_C_6_H_4_-*m*F | CH_2_-CHBr-C_6_H_5_ | 9.54 | 2.556 | 0.331 | 0.318 |
| 14 | SCH_3_ | NHC_6_H_4_-*p*F | CH_2_-CHCl-C_6_H_5_ | 1.778 | 2.229 | 0.234 | 0.251 |
| 15 | SCH_3_ | NHC_6_H_4_-*o*F | CH_2_-CHF-C_6_H_5_-pF | 2.454 | 2.402 | 0.132 | 0.184 |
| 16 | SCH_3_ | NHCH_2_C_6_H_4_-*m*F | CH_2_-CHF-C_6_H_5_-pF | 1.097 | 1.113 | 0.389 | 0.207 |
| 17 | SCH_3_ | NHC_6_H_4_-*p*F | CH_2_-CHBr-C_6_H_5_-pF | 0.851 | 0.919 | 0.007 | 0.001 |
| 18 | SCH_3_ | NHCH_2_C_6_H_4_-*m*F | CH_2_-CHCl-C_6_H_5_ | 1.738 | 2.645 | 0.324 | 0.378 |
| 19 | SCH_3_ | NHCH_2_CH_2_C_6_H_4_-*m*F | CH_2_-CHCl-C_6_H_5_ | 1.514 | 2.512 | 0.234 | 0.305 |
| 20 | SCH_3_ | NHCH_2_CH_2_C_6_H_4_-*m*F | CH_2_-CHF-C_6_H_5_ | 3.02 | 1.574 | 0.178, 0.708 | 0.35 |
