# Supplementary material for: Optimized Hydrophobic Interactions and Hydrogen Bonding at the Target-Ligand Interface Leads the Pathways of Drug-Designing
Source: PLoS One. 2010 Aug 16;5(8):e12029. doi: 10.1371/journal.pone.0012029 (PMC2922327; doi:10.1371/journal.pone.0012029)
Supplement: Table S3 — Cavity volume in the protein structures of c-Src and c-Abl selected for docking. (0.03 MB DOC) [file pone.0012029.s004.doc]

**Table S3: Cavity volume in the protein structures of c-Src and c-Abl selected for docking**

| Protein Name | PDB ID | Resolution ( Å) | Binding Site Volume (Å^3^) |
| --- | --- | --- | --- |
| c-Src | 1YOL | 2.3 | 243.875 |
|  | 1FMK | 1.5 | 338.125 |
|  | 2H8H | 2.2 | 295.625 |
| c-Abl | 1M52 | 2.6 | 315.25 |
|  | 2FO0 | 2.27 | 262.000 |
