# Supplementary material for: Optimized Hydrophobic Interactions and Hydrogen Bonding at the Target-Ligand Interface Leads the Pathways of Drug-Designing
Source: PLoS One. 2010 Aug 16;5(8):e12029. doi: 10.1371/journal.pone.0012029 (PMC2922327; doi:10.1371/journal.pone.0012029)
Supplement: Table S4 — RMSD values of ligands (only for the molecules 1, 12, 19 and 28) at binding site of c-Src and c-Abl measured using different algorithms. (0.04 MB DOC) [file pone.0012029.s005.doc]

**Table S4: RMSD values of ligands (only for the molecules 1, 12, 19 and 28) at binding site of c-Src and c-Abl measured using different algorithms**

| Molecules | | LigandFit-ZDOCK | LigandFit-CDOCKER | ZDOCK-CDOCKER |
| --- | --- | --- | --- | --- |
| Src | 1 | 0.5600 | 0.1408 | 0.545 |
|  | 12 | 0.8962 | 0.1781 | 0.8545 |
|  | 19 | 0.5451 | 0.1601 | 0.4961 |
|  | 28 | 0.4093 | 0.8603 | 0.8685 |
| Abl | 1 | 0.9164 | 0.3141 | 1.0745 |
|  | 12 | 0.5296 | 0.9292 | 0.8167 |
|  | 19 | 0.6847 | 0.8748 | 0.4985 |
|  | 28 | 0.7731 | 0.1892 | 0.7989 |
